# Supplementary material for: Mutual interaction between motor cortex activation and pain in fibromyalgia: EEG-fNIRS study
Source: PLoS One. 2020 Jan 23;15(1):e0228158. doi: 10.1371/journal.pone.0228158 (PMC6977766; doi:10.1371/journal.pone.0228158)
Supplement: S1 Table — (DOCX) [file pone.0228158.s001.docx]

**S1 Table. Latency and Amplitude of LEP components for patients’ group.**

|  | Right Hand Stimulation | | Left Hand Stimulation | |
| --- | --- | --- | --- | --- |
|  | Mean | Std.  deviation | Mean | Std.  Deviation |
| N1 LATENCY | 0,177 (s) | 0,117 | 0,175 (s) | 0,133 |
| N1 AMPLITUDE | -3,67 (µV) | 43,95 | -1,77 (µV)* | 36,10 |
| N2 LATENCY | 0,223 (s) | 0,196 | 0,226 (s) | 0,180 |
| P2 LATENCY | 0,330 (s) | 0,223 | 0,322 (s) | 0,187 |
| N2-P2 AMPLITUDE | 14,98 (µV) | 72,10 | 14,96 (µV) | 66,06 |
| N1 LATENCY  DURING SFT | 0,178 (s) | 0,151 | 0,170 (s) | 0,139 |
| N1 AMPLITUDE  DURING SFT | -3,41 (µV)* | 31,09 | -4,36 (µV) | 38,98 |
| N2 LATENCY  DURING SFT | 0,231 (s) | 0,241 | 0,230 (s) | 0,236 |
| P2 LATENCY  DURING SFT | 0,332 (s) | 0,301 | 0,329 (s) | 0,237 |
| N2-P2 AMPLITUDE  DURING SFT | 12,56 (µV) | 51,31 | 13,19 (µV) | 43,51 |
| N1 LATENCY  DURING FFT | 0,174(s) | 0,113 | 0,179 (s) | 0,110 |
| N1 AMPLITUDE  DURING FFT | -1,45 (µV) | 41,68 | -4,01 (µV) | 45,06 |
| N2 LATENCY DURING FFT | 0,225 (s) | 0,194 | 0,236 (s) | 0,245 |
| P2 LATENCY  DURING FFT | 0,327 (s) | 0,195 | 0,335 (s) | 0,215 |
| N2-P2 AMPLITUDE  DURING FFT | 12,91 (µV) | 58,83 | 14,97 (µV) | 62,09 |
