# Supplementary material for: Mutual interaction between motor cortex activation and pain in fibromyalgia: EEG-fNIRS study
Source: PLoS One. 2020 Jan 23;15(1):e0228158. doi: 10.1371/journal.pone.0228158 (PMC6977766; doi:10.1371/journal.pone.0228158)
Supplement: S2 Table — (DOCX) [file pone.0228158.s002.docx]

**S2 Table**. **Latency and Amplitude of LEP components for controls’ group.**

|  | Right Hand Stimulation | | Left Hand Stimulation | |
| --- | --- | --- | --- | --- |
|  | Mean | Std. Deviation | Mean | Std. Deviation |
| N1 LATENCY | 0,178 (s) | 0,090 | 0,173 (s) | 0,078 |
| N1 AMPLITUDE | -7,56 (µV) | 52,70 | -4,83 (µV)* | 38,83 |
| N2 LATENCY | 0,221 (s) | 0,126 | 0,231 (s) | 0,122 |
| P2 LATENCY | 0,339 (s) | 0,222 | 0,341 (s) | 0,197 |
| N2-P2 AMPLITUDE | 18,93 (µV) | 48,61 | 17, 62 (µV) | 62,91 |
| N1 LATENCY  DURING SFT | 0,173 (s) | 0,099 | 0,171 (s) | 0,083 |
| N1 AMPLITUDE  DURING SFT | -7,36 (µV)* | 32,74 | -2,43 (µV) | 42,92 |
| N2 LATENCY  DURING SFT | 0,224 (s) | 0,160 | 0,232 (s) | 0,219 |
| P2 LATENCY  DURING SFT | 0,350 (s) | 0,183 | 0,352 (s) | 0,202 |
| N2-P2 AMPLITUDE  DURING SFT | 18,64 (µV) | 69,32 | 15,07 (µV) | 43,58 |
| N1 LATENCY  DURING FFT | 0,186 (s) | 0,061 | 0,176 (s) | 0,078 |
| N1 AMPLITUDE  DURING FFT | -3,32 (µV) | 61,39 | -4,28 (µV) | 48,24 |
| N2 LATENCY  DURING FFT | 0,229 (s) | 0,214 | 0,230 (s) | 0,194 |
| P2 LATENCY  DURING FFT | 0,352 (s) | 0,187 | 0,342 (s) | 0,246 |
| N2-P2 AMPLITUDE  DURING FFT | 19,62 (µV) | 58,54 | 14,97 (µV) | 54,52 |
