# Supplementary material for: Mutual interaction between motor cortex activation and pain in fibromyalgia: EEG-fNIRS study
Source: PLoS One. 2020 Jan 23;15(1):e0228158. doi: 10.1371/journal.pone.0228158 (PMC6977766; doi:10.1371/journal.pone.0228158)
Supplement: S3 Table — (DOCX) [file pone.0228158.s003.docx]

**S3 Table. Correlations for FFT + LASER ON THE RIGHT HAND condition.**

| **Correlations in FFT + LASER ON THE RIGHT HAND** | | | | | | |
| --- | --- | --- | --- | --- | --- | --- |
|  |  | Clinical Variable | | | | |
|  |  | sas | sds | maf | Disease Duration  (years) | wPi |
| Channel_1 | Pearson Correlation | -,292^*^ | -.207 | -.168 | .000 | -.443 |
|  | Sig. (2-tailed) | .038 | .145 | .238 | .997 | .455 |
|  | N | 51 | 51 | 51 | 46 | 5 |
| Channel_2 | Pearson Correlation | -,286^*^ | -.265 | -.113 | .013 | -.629 |
|  | Sig. (2-tailed) | .042 | .060 | .431 | .933 | .256 |
|  | N | 51 | 51 | 51 | 46 | 5 |
| Channel_3 | Pearson Correlation | -.252 | -.218 | -.050 | -.008 | -.494 |
|  | Sig. (2-tailed) | .078 | .128 | .729 | .959 | .398 |
|  | N | 50 | 50 | 50 | 45 | 5 |
| Channel_4 | Pearson Correlation | -,309^*^ | -,298^*^ | -.203 | -.091 | -.469 |
|  | Sig. (2-tailed) | .027 | .034 | .153 | .548 | .426 |
|  | N | 51 | 51 | 51 | 46 | 5 |
| Channel_5 | Pearson Correlation | -,307^*^ | -.248 | -.056 | -.097 | -.399 |
|  | Sig. (2-tailed) | .028 | .079 | .695 | .521 | .505 |
|  | N | 51 | 51 | 51 | 46 | 5 |
| Channel_6 | Pearson Correlation | -.225 | -.225 | -.087 | -.102 | -.824 |
|  | Sig. (2-tailed) | .113 | .113 | .545 | .501 | .086 |
|  | N | 51 | 51 | 51 | 46 | 5 |
| Channel_7 | Pearson Correlation | -.195 | -.163 | .022 | .077 | -.706 |
|  | Sig. (2-tailed) | .175 | .257 | .880 | .614 | .183 |
|  | N | 50 | 50 | 50 | 45 | 5 |
| Channel_8 | Pearson Correlation | -.110 | -.096 | .064 | .030 | -.715 |
|  | Sig. (2-tailed) | .440 | .503 | .656 | .844 | .175 |
|  | N | 51 | 51 | 51 | 46 | 5 |
| Channel_9 | Pearson Correlation | .115 | .029 | .081 | .211 | -.656 |
|  | Sig. (2-tailed) | .425 | .840 | .576 | .164 | .230 |
|  | N | 50 | 50 | 50 | 45 | 5 |
| Channel_10 | Pearson Correlation | -.016 | -.158 | -.081 | -.077 | -.833 |
|  | Sig. (2-tailed) | .913 | .277 | .581 | .621 | .080 |
|  | N | 49 | 49 | 49 | 44 | 5 |
| Channel_11 | Pearson Correlation | -,283^*^ | -.178 | -.025 | -.012 | -.667 |
|  | Sig. (2-tailed) | .046 | .217 | .863 | .937 | .219 |
|  | N | 50 | 50 | 50 | 45 | 5 |
| Channel_12 | Pearson Correlation | -.193 | -.139 | -.090 | .052 | -.676 |
|  | Sig. (2-tailed) | .184 | .341 | .539 | .737 | .210 |
|  | N | 49 | 49 | 49 | 44 | 5 |
| Channel_13 | Pearson Correlation | -.121 | -.129 | -.093 | -.047 | -.647 |
|  | Sig. (2-tailed) | .402 | .371 | .523 | .758 | .238 |
|  | N | 50 | 50 | 50 | 45 | 5 |
| Channel_14 | Pearson Correlation | -.283 | -.243 | -.190 | -.032 | -.806 |
|  | Sig. (2-tailed) | .051 | .096 | .196 | .837 | .099 |
|  | N | 48 | 48 | 48 | 44 | 5 |
| Channel_15 | Pearson Correlation | -.014 | -.061 | -.092 | -.080 | -.599 |
|  | Sig. (2-tailed) | .925 | .672 | .527 | .601 | .285 |
|  | N | 50 | 50 | 50 | 45 | 5 |
| Channel_16 | Pearson Correlation | -.112 | -.092 | -.032 | .084 | .294 |
|  | Sig. (2-tailed) | .443 | .530 | .827 | .589 | .631 |
|  | N | 49 | 49 | 49 | 44 | 5 |
| Channel_17 | Pearson Correlation | -.117 | -.080 | .029 | -.034 | -.540 |
|  | Sig. (2-tailed) | .424 | .586 | .843 | .827 | .347 |
|  | N | 49 | 49 | 49 | 44 | 5 |
| Channel_18 | Pearson Correlation | -.064 | -.092 | -.133 | -.074 | -.590 |
|  | Sig. (2-tailed) | .658 | .520 | .353 | .626 | .295 |
|  | N | 51 | 51 | 51 | 46 | 5 |
| Channel_19 | Pearson Correlation | -.188 | -.247 | -,308^*^ | -.086 | -.386 |
|  | Sig. (2-tailed) | .197 | .086 | .031 | .578 | .521 |
|  | N | 49 | 49 | 49 | 44 | 5 |
| Channel_20 | Pearson Correlation | -.057 | -.081 | -.129 | -.060 | -.564 |
|  | Sig. (2-tailed) | .693 | .576 | .371 | .698 | .322 |
|  | N | 50 | 50 | 50 | 45 | 5 |
| Channel_1  deoxy | Pearson Correlation | -.042 | .197 | .136 | .099 | -.370 |
|  | Sig. (2-tailed) | .770 | .166 | .341 | .514 | .540 |
|  | N | 51 | 51 | 51 | 46 | 5 |
| Channel_2  deoxy | Pearson Correlation | .131 | ,298^*^ | .227 | -.015 | -.515 |
|  | Sig. (2-tailed) | .359 | .033 | .109 | .920 | .374 |
|  | N | 51 | 51 | 51 | 46 | 5 |
| Channel_3  deoxy | Pearson Correlation | -.204 | -.007 | -.271 | -.139 | -.722 |
|  | Sig. (2-tailed) | .156 | .962 | .057 | .362 | .168 |
|  | N | 50 | 50 | 50 | 45 | 5 |
| Channel_4  deoxy | Pearson Correlation | .052 | .196 | .100 | -.074 | .060 |
|  | Sig. (2-tailed) | .715 | .168 | .486 | .627 | .923 |
|  | N | 51 | 51 | 51 | 46 | 5 |
| Channel_5  deoxy | Pearson Correlation | -.250 | -.075 | -.245 | -.144 | .046 |
|  | Sig. (2-tailed) | .077 | .599 | .083 | .340 | .941 |
|  | N | 51 | 51 | 51 | 46 | 5 |
| Channel_6  deoxy | Pearson Correlation | -.235 | -.237 | -.249 | -.130 | .593 |
|  | Sig. (2-tailed) | .097 | .094 | .078 | .388 | .292 |
|  | N | 51 | 51 | 51 | 46 | 5 |
| Channel_7  deoxy | Pearson Correlation | .083 | .168 | .017 | .106 | -.378 |
|  | Sig. (2-tailed) | .567 | .245 | .908 | .487 | .530 |
|  | N | 50 | 50 | 50 | 45 | 5 |
| Channel_8  deoxy | Pearson Correlation | .007 | .025 | -.106 | -.007 | -.378 |
|  | Sig. (2-tailed) | .961 | .860 | .458 | .964 | .530 |
|  | N | 51 | 51 | 51 | 46 | 5 |
| Channel_9  deoxy | Pearson Correlation | -,279^*^ | -,296^*^ | -,294^*^ | -.185 | .249 |
|  | Sig. (2-tailed) | .050 | .037 | .038 | .223 | .686 |
|  | N | 50 | 50 | 50 | 45 | 5 |
| Channel_10  deoxy | Pearson Correlation | .052 | .125 | -.023 | -.018 | ,990^**^ |
|  | Sig. (2-tailed) | .723 | .393 | .874 | .907 | .001 |
|  | N | 49 | 49 | 49 | 44 | 5 |
| Channel_11  deoxy | Pearson Correlation | -.055 | .051 | -.054 | .051 | -.249 |
|  | Sig. (2-tailed) | .707 | .726 | .712 | .738 | .686 |
|  | N | 50 | 50 | 50 | 45 | 5 |
| Channel_12  deoxy | Pearson Correlation | -.030 | -.017 | -.043 | .073 | .473 |
|  | Sig. (2-tailed) | .838 | .908 | .771 | .640 | .421 |
|  | N | 49 | 49 | 49 | 44 | 5 |
| Channel_13  deoxy | Pearson Correlation | -.214 | -.134 | -.166 | -.164 | -.477 |
|  | Sig. (2-tailed) | .136 | .353 | .250 | .281 | .417 |
|  | N | 50 | 50 | 50 | 45 | 5 |
| Channel_14  deoxy | Pearson Correlation | -.257 | -.097 | -.026 | -.236 | -.832 |
|  | Sig. (2-tailed) | .078 | .514 | .862 | .124 | .080 |
|  | N | 48 | 48 | 48 | 44 | 5 |
| Channel_15  deoxy | Pearson Correlation | .079 | .071 | -.173 | -.091 | -.154 |
|  | Sig. (2-tailed) | .588 | .622 | .229 | .551 | .804 |
|  | N | 50 | 50 | 50 | 45 | 5 |
| Channel_16  deoxy | Pearson Correlation | -.112 | -.086 | -.022 | .197 | -.477 |
|  | Sig. (2-tailed) | .442 | .559 | .879 | .200 | .417 |
|  | N | 49 | 49 | 49 | 44 | 5 |
| Channel_17  deoxy | Pearson Correlation | .055 | .058 | .108 | .009 | .498 |
|  | Sig. (2-tailed) | .706 | .692 | .461 | .952 | .394 |
|  | N | 49 | 49 | 49 | 44 | 5 |
| Channel_18  deoxy | Pearson Correlation | .108 | .095 | -.078 | -.005 | -.551 |
|  | Sig. (2-tailed) | .449 | .506 | .585 | .973 | .336 |
|  | N | 51 | 51 | 51 | 46 | 5 |
| Channel_19  deoxy | Pearson Correlation | -.002 | .118 | -.220 | -.104 | .122 |
|  | Sig. (2-tailed) | .989 | .419 | .128 | .503 | .845 |
|  | N | 49 | 49 | 49 | 44 | 5 |
| Channel_20  deoxy | Pearson Correlation | -.053 | -.073 | -.116 | .204 | -.559 |
|  | Sig. (2-tailed) | .714 | .615 | .421 | .179 | .327 |
|  | N | 50 | 50 | 50 | 45 | 5 |

*. Correlation is significant at the 0.05 level (2-tailed).

**. Correlation is significant at the 0.01 level (2-tailed).
