# Supplementary material for: Mutual interaction between motor cortex activation and pain in fibromyalgia: EEG-fNIRS study
Source: PLoS One. 2020 Jan 23;15(1):e0228158. doi: 10.1371/journal.pone.0228158 (PMC6977766; doi:10.1371/journal.pone.0228158)
Supplement: S4 Table — (DOCX) [file pone.0228158.s004.docx]

**S4 Table. Correlations for FFT + LASER ON THE LEFT HAND condition.**

| **Correlations in FFT + LASER ON THE LEFT HAND** | | | | | | |
| --- | --- | --- | --- | --- | --- | --- |
|  |  | Clinical Variable | | | | |
|  |  | sas | sds | maf | Disease Duration  (years) | wPi |
| Channel_1 | Pearson Correlation | -.107 | -.147 | -.225 | .078 | -.007 |
|  | Sig. (2-tailed) | .454 | .305 | .112 | .608 | .992 |
|  | N | 51 | 51 | 51 | 46 | 5 |
| Channel_2 | Pearson Correlation | -.204 | -.202 | -.151 | .021 | -.102 |
|  | Sig. (2-tailed) | .152 | .155 | .291 | .888 | .870 |
|  | N | 51 | 51 | 51 | 46 | 5 |
| Channel_3 | Pearson Correlation | -.240 | -.259 | -.096 | -.017 | .475 |
|  | Sig. (2-tailed) | .093 | .070 | .509 | .910 | .419 |
|  | N | 50 | 50 | 50 | 45 | 5 |
| Channel_4 | Pearson Correlation | -,329^*^ | -,364^**^ | -,299^*^ | -.193 | -.293 |
|  | Sig. (2-tailed) | .019 | .009 | .033 | .198 | .632 |
|  | N | 51 | 51 | 51 | 46 | 5 |
| Channel_5 | Pearson Correlation | -,309^*^ | -,297^*^ | -.121 | -.143 | .351 |
|  | Sig. (2-tailed) | .027 | .034 | .396 | .342 | .563 |
|  | N | 51 | 51 | 51 | 46 | 5 |
| Channel_6 | Pearson Correlation | -,285^*^ | -,279^*^ | -.152 | -.207 | .379 |
|  | Sig. (2-tailed) | .043 | .048 | .286 | .168 | .529 |
|  | N | 51 | 51 | 51 | 46 | 5 |
| Channel_7 | Pearson Correlation | -.092 | -.141 | -.060 | .068 | -.366 |
|  | Sig. (2-tailed) | .526 | .329 | .681 | .656 | .544 |
|  | N | 50 | 50 | 50 | 45 | 5 |
| Channel_8 | Pearson Correlation | -.149 | -.219 | -.090 | -.040 | -.132 |
|  | Sig. (2-tailed) | .297 | .123 | .530 | .792 | .833 |
|  | N | 51 | 51 | 51 | 46 | 5 |
| Channel_9 | Pearson Correlation | .042 | -.080 | -.017 | .108 | .677 |
|  | Sig. (2-tailed) | .770 | .580 | .907 | .482 | .209 |
|  | N | 50 | 50 | 50 | 45 | 5 |
| Channel_10 | Pearson Correlation | -.210 | -.279 | -.121 | -.262 | .488 |
|  | Sig. (2-tailed) | .148 | .052 | .409 | .085 | .404 |
|  | N | 49 | 49 | 49 | 44 | 5 |
| Channel_11 | Pearson Correlation | -.210 | -.161 | -.186 | .020 | .000 |
|  | Sig. (2-tailed) | .144 | .265 | .195 | .896 | 1.000 |
|  | N | 50 | 50 | 50 | 45 | 5 |
| Channel_12 | Pearson Correlation | -.196 | -.165 | -.176 | .065 | -.329 |
|  | Sig. (2-tailed) | .178 | .256 | .228 | .675 | .589 |
|  | N | 49 | 49 | 49 | 44 | 5 |
| Channel_13 | Pearson Correlation | -.033 | -.131 | -.114 | .010 | -.476 |
|  | Sig. (2-tailed) | .822 | .366 | .431 | .950 | .418 |
|  | N | 50 | 50 | 50 | 45 | 5 |
| Channel_14 | Pearson Correlation | -,344^*^ | -.284 | -.214 | -.106 | -.204 |
|  | Sig. (2-tailed) | .017 | .051 | .145 | .494 | .742 |
|  | N | 48 | 48 | 48 | 44 | 5 |
| Channel_15 | Pearson Correlation | -.076 | -.174 | -.126 | -.124 | -.547 |
|  | Sig. (2-tailed) | .599 | .227 | .383 | .416 | .340 |
|  | N | 50 | 50 | 50 | 45 | 5 |
| Channel_16 | Pearson Correlation | -.131 | -.114 | -.040 | -.234 | .530 |
|  | Sig. (2-tailed) | .370 | .436 | .786 | .127 | .358 |
|  | N | 49 | 49 | 49 | 44 | 5 |
| Channel_17 | Pearson Correlation | -.073 | -.130 | -.059 | .006 | -.564 |
|  | Sig. (2-tailed) | .617 | .372 | .689 | .968 | .322 |
|  | N | 49 | 49 | 49 | 44 | 5 |
| Channel_18 | Pearson Correlation | -.039 | -.165 | -.180 | -.042 | -.419 |
|  | Sig. (2-tailed) | .785 | .247 | .208 | .780 | .483 |
|  | N | 51 | 51 | 51 | 46 | 5 |
| Channel_19 | Pearson Correlation | -.168 | -.269 | -,318^*^ | -.191 | -.019 |
|  | Sig. (2-tailed) | .249 | .061 | .026 | .214 | .976 |
|  | N | 49 | 49 | 49 | 44 | 5 |
| Channel_20 | Pearson Correlation | -.065 | -.094 | -.146 | -,332^*^ | -.579 |
|  | Sig. (2-tailed) | .652 | .516 | .312 | .026 | .306 |
|  | N | 50 | 50 | 50 | 45 | 5 |
| Channel_1  deoxy | Pearson Correlation | .080 | .110 | -.010 | .118 | -.530 |
|  | Sig. (2-tailed) | .578 | .444 | .947 | .435 | .359 |
|  | N | 51 | 51 | 51 | 46 | 5 |
| Channel_2  deoxy | Pearson Correlation | .109 | .092 | .175 | -.015 | .401 |
|  | Sig. (2-tailed) | .445 | .523 | .218 | .923 | .503 |
|  | N | 51 | 51 | 51 | 46 | 5 |
| Channel_3  deoxy | Pearson Correlation | -.154 | -.157 | -,310^*^ | -.075 | -.121 |
|  | Sig. (2-tailed) | .284 | .276 | .029 | .625 | .846 |
|  | N | 50 | 50 | 50 | 45 | 5 |
| Channel_4  deoxy | Pearson Correlation | -.098 | -.037 | -.053 | -.089 | .332 |
|  | Sig. (2-tailed) | .494 | .796 | .714 | .555 | .585 |
|  | N | 51 | 51 | 51 | 46 | 5 |
| Channel_5  deoxy | Pearson Correlation | -,281^*^ | -.159 | -.260 | -.122 | .482 |
|  | Sig. (2-tailed) | .046 | .265 | .066 | .418 | .411 |
|  | N | 51 | 51 | 51 | 46 | 5 |
| Channel_6  deoxy | Pearson Correlation | -,287^*^ | -,333^*^ | -.223 | -.137 | .335 |
|  | Sig. (2-tailed) | .041 | .017 | .117 | .363 | .581 |
|  | N | 51 | 51 | 51 | 46 | 5 |
| Channel_7  deoxy | Pearson Correlation | .168 | .016 | -.060 | .217 | .619 |
|  | Sig. (2-tailed) | .244 | .911 | .680 | .152 | .266 |
|  | N | 50 | 50 | 50 | 45 | 5 |
| Channel_8  deoxy | Pearson Correlation | -.014 | -.094 | -.131 | -.082 | .173 |
|  | Sig. (2-tailed) | .920 | .514 | .358 | .587 | .781 |
|  | N | 51 | 51 | 51 | 46 | 5 |
| Channel_9  deoxy | Pearson Correlation | -.078 | -.080 | -.063 | -.092 | .553 |
|  | Sig. (2-tailed) | .588 | .580 | .663 | .549 | .334 |
|  | N | 50 | 50 | 50 | 45 | 5 |
| Channel_10  deoxy | Pearson Correlation | -.124 | -.100 | -.069 | -.183 | .584 |
|  | Sig. (2-tailed) | .398 | .493 | .636 | .236 | .301 |
|  | N | 49 | 49 | 49 | 44 | 5 |
| Channel_11  deoxy | Pearson Correlation | -.109 | -.109 | -.076 | -.024 | .561 |
|  | Sig. (2-tailed) | .452 | .451 | .599 | .877 | .325 |
|  | N | 50 | 50 | 50 | 45 | 5 |
| Channel_12  deoxy | Pearson Correlation | .058 | .075 | .008 | .081 | .623 |
|  | Sig. (2-tailed) | .690 | .610 | .956 | .600 | .262 |
|  | N | 49 | 49 | 49 | 44 | 5 |
| Channel_13  deoxy | Pearson Correlation | .014 | -.040 | -.050 | -.095 | .525 |
|  | Sig. (2-tailed) | .921 | .785 | .732 | .533 | .364 |
|  | N | 50 | 50 | 50 | 45 | 5 |
| Channel_14  deoxy | Pearson Correlation | -.244 | -.207 | .007 | -.100 | -.095 |
|  | Sig. (2-tailed) | .094 | .158 | .960 | .517 | .879 |
|  | N | 48 | 48 | 48 | 44 | 5 |
| Channel_15  deoxy | Pearson Correlation | .151 | .026 | -.055 | -.062 | .312 |
|  | Sig. (2-tailed) | .294 | .858 | .707 | .686 | .609 |
|  | N | 50 | 50 | 50 | 45 | 5 |
| Channel_16  deoxy | Pearson Correlation | -.111 | -.089 | -.019 | .051 | -.245 |
|  | Sig. (2-tailed) | .446 | .541 | .897 | .742 | .692 |
|  | N | 49 | 49 | 49 | 44 | 5 |
| Channel_17  deoxy | Pearson Correlation | .052 | -.032 | .049 | .033 | .515 |
|  | Sig. (2-tailed) | .722 | .829 | .739 | .830 | .374 |
|  | N | 49 | 49 | 49 | 44 | 5 |
| Channel_18  deoxy | Pearson Correlation | .124 | -.032 | -.041 | -.004 | -.598 |
|  | Sig. (2-tailed) | .387 | .824 | .776 | .981 | .287 |
|  | N | 51 | 51 | 51 | 46 | 5 |
| Channel_19  deoxy | Pearson Correlation | .054 | .070 | -.119 | -.121 | .368 |
|  | Sig. (2-tailed) | .714 | .633 | .414 | .433 | .542 |
|  | N | 49 | 49 | 49 | 44 | 5 |
| Channel_20  deoxy | Pearson Correlation | -.069 | -.083 | -.118 | -.010 | -.553 |
|  | Sig. (2-tailed) | .635 | .565 | .416 | .950 | .333 |
|  | N | 50 | 50 | 50 | 45 | 5 |

*. Correlation is significant at the 0.05 level (2-tailed).

**. Correlation is significant at the 0.01 level (2-tailed).
