# Supplementary material for: Mutual interaction between motor cortex activation and pain in fibromyalgia: EEG-fNIRS study
Source: PLoS One. 2020 Jan 23;15(1):e0228158. doi: 10.1371/journal.pone.0228158 (PMC6977766; doi:10.1371/journal.pone.0228158)
Supplement: S5 Table — (DOCX) [file pone.0228158.s005.docx]

**S5 Table. Correlations for FFT condition**.

| **Correlations in FFT** | | | | | | | |
| --- | --- | --- | --- | --- | --- | --- | --- |
|  |  | Clinical Variable | | | | |  |
|  |  | sas | sds | maf | Disease Duration  (years) | wPi |  |
| Channel_1 | Pearson Correlation | -.201 | -.214 | -.187 | .003 | -.676 |  |
|  | Sig. (2-tailed) | .157 | .131 | .190 | .982 | .211 |  |
|  | N | 51 | 51 | 51 | 46 | 5 |  |
| Channel_2 | Pearson Correlation | -.268 | -.217 | -.066 | -.045 | -.849 |  |
|  | Sig. (2-tailed) | .058 | .126 | .644 | .766 | .069 |  |
|  | N | 51 | 51 | 51 | 46 | 5 |  |
| Channel_3 | Pearson Correlation | -.233 | -.214 | -.046 | -.059 | -.813 |  |
|  | Sig. (2-tailed) | .104 | .136 | .753 | .701 | .094 |  |
|  | N | 50 | 50 | 50 | 45 | 5 |  |
| Channel_4 | Pearson Correlation | -,327^*^ | -,305^*^ | -.234 | -.185 | -.745 |  |
|  | Sig. (2-tailed) | .019 | .029 | .099 | .219 | .148 |  |
|  | N | 51 | 51 | 51 | 46 | 5 |  |
| Channel_5 | Pearson Correlation | -,346^*^ | -,279^*^ | -.092 | -.193 | -.609 |  |
|  | Sig. (2-tailed) | .013 | .048 | .521 | .199 | .275 |  |
|  | N | 51 | 51 | 51 | 46 | 5 |  |
| Channel_6 | Pearson Correlation | -,356^*^ | -,365^**^ | -.163 | -.222 | -.802 |  |
|  | Sig. (2-tailed) | .010 | .009 | .252 | .138 | .102 |  |
|  | N | 51 | 51 | 51 | 46 | 5 |  |
| Channel_7 | Pearson Correlation | -.184 | -.152 | .019 | -.044 | -.761 |  |
|  | Sig. (2-tailed) | .202 | .293 | .895 | .773 | .135 |  |
|  | N | 50 | 50 | 50 | 45 | 5 |  |
| Channel_8 | Pearson Correlation | -.161 | -.155 | -.035 | -.085 | -.771 |  |
|  | Sig. (2-tailed) | .259 | .278 | .809 | .572 | .127 |  |
|  | N | 51 | 51 | 51 | 46 | 5 |  |
| Channel_9 | Pearson Correlation | -.139 | -.240 | -.174 | -.069 | -.798 |  |
|  | Sig. (2-tailed) | .337 | .094 | .228 | .652 | .105 |  |
|  | N | 50 | 50 | 50 | 45 | 5 |  |
| Channel_10 | Pearson Correlation | -.183 | -,369^**^ | -.227 | -.244 | -.287 |  |
|  | Sig. (2-tailed) | .209 | .009 | .117 | .111 | .640 |  |
|  | N | 49 | 49 | 49 | 44 | 5 |  |
| Channel_11 | Pearson Correlation | -.191 | -.145 | -.042 | -.028 | -.761 |  |
|  | Sig. (2-tailed) | .184 | .315 | .773 | .856 | .135 |  |
|  | N | 50 | 50 | 50 | 45 | 5 |  |
| Channel_12 | Pearson Correlation | -.278 | -.237 | -.108 | -.001 | -.621 |  |
|  | Sig. (2-tailed) | .053 | .101 | .459 | .995 | .263 |  |
|  | N | 49 | 49 | 49 | 44 | 5 |  |
| Channel_13 | Pearson Correlation | -.113 | -.183 | -.115 | -.067 | -.729 |  |
|  | Sig. (2-tailed) | .433 | .202 | .425 | .663 | .162 |  |
|  | N | 50 | 50 | 50 | 45 | 5 |  |
| Channel_14 | Pearson Correlation | -.267 | -.207 | -.091 | -.059 | -.731 |  |
|  | Sig. (2-tailed) | .066 | .158 | .539 | .705 | .160 |  |
|  | N | 48 | 48 | 48 | 44 | 5 |  |
| Channel_15 | Pearson Correlation | -.088 | -.108 | -.075 | -.154 | -.706 |  |
|  | Sig. (2-tailed) | .546 | .457 | .607 | .313 | .183 |  |
|  | N | 50 | 50 | 50 | 45 | 5 |  |
| Channel_16 | Pearson Correlation | -.155 | -.130 | -.043 | -.292 | -.697 |  |
|  | Sig. (2-tailed) | .288 | .375 | .768 | .054 | .191 |  |
|  | N | 49 | 49 | 49 | 44 | 5 |  |
| Channel_17 | Pearson Correlation | -.192 | -.182 | -.007 | -.063 | -.672 |  |
|  | Sig. (2-tailed) | .187 | .211 | .964 | .683 | .214 |  |
|  | N | 49 | 49 | 49 | 44 | 5 |  |
| Channel_18 | Pearson Correlation | -.143 | -.211 | -.195 | -.134 | -.684 |  |
|  | Sig. (2-tailed) | .317 | .137 | .170 | .373 | .203 |  |
|  | N | 51 | 51 | 51 | 46 | 5 |  |
| Channel_19 | Pearson Correlation | -,290^*^ | -,354^*^ | -,308^*^ | -.269 | -.682 |  |
|  | Sig. (2-tailed) | .044 | .012 | .031 | .077 | .204 |  |
|  | N | 49 | 49 | 49 | 44 | 5 |  |
| Channel_20 | Pearson Correlation | -.148 | -.214 | -.257 | -,392^**^ | -.598 |  |
|  | Sig. (2-tailed) | .305 | .137 | .071 | .008 | .287 |  |
|  | N | 50 | 50 | 50 | 45 | 5 |  |
| Channel_1  deoxy | Pearson Correlation | .105 | ,287^*^ | .080 | .249 | -.575 |  |
|  | Sig. (2-tailed) | .463 | .041 | .576 | .095 | .311 |  |
|  | N | 51 | 51 | 51 | 46 | 5 |  |
| Channel_2  deoxy | Pearson Correlation | .158 | ,355^*^ | .195 | .006 | -.583 |  |
|  | Sig. (2-tailed) | .268 | .011 | .170 | .968 | .302 |  |
|  | N | 51 | 51 | 51 | 46 | 5 |  |
| Channel_3  deoxy | Pearson Correlation | -.072 | .082 | -.181 | -.009 | -.607 |  |
|  | Sig. (2-tailed) | .618 | .573 | .208 | .951 | .278 |  |
|  | N | 50 | 50 | 50 | 45 | 5 |  |
| Channel_4  deoxy | Pearson Correlation | .072 | .160 | .048 | .016 | -.279 |  |
|  | Sig. (2-tailed) | .616 | .261 | .738 | .915 | .650 |  |
|  | N | 51 | 51 | 51 | 46 | 5 |  |
| Channel_5  deoxy | Pearson Correlation | -.249 | -.075 | -.223 | -.098 | -.400 |  |
|  | Sig. (2-tailed) | .078 | .600 | .116 | .518 | .504 |  |
|  | N | 51 | 51 | 51 | 46 | 5 |  |
| Channel_6  deoxy | Pearson Correlation | -.244 | -.252 | -.238 | -.115 | .304 |  |
|  | Sig. (2-tailed) | .084 | .075 | .093 | .446 | .620 |  |
|  | N | 51 | 51 | 51 | 46 | 5 |  |
| Channel_7  deoxy | Pearson Correlation | .093 | .216 | .027 | .028 | -.618 |  |
|  | Sig. (2-tailed) | .522 | .132 | .851 | .857 | .267 |  |
|  | N | 50 | 50 | 50 | 45 | 5 |  |
| Channel_8  deoxy | Pearson Correlation | -.030 | .028 | -.100 | -.059 | -.333 |  |
|  | Sig. (2-tailed) | .836 | .845 | .484 | .695 | .584 |  |
|  | N | 51 | 51 | 51 | 46 | 5 |  |
| Channel_9  deoxy | Pearson Correlation | -.100 | -.033 | -.032 | -.117 | -.525 |  |
|  | Sig. (2-tailed) | .489 | .819 | .828 | .443 | .364 |  |
|  | N | 50 | 50 | 50 | 45 | 5 |  |
| Channel_10  deoxy | Pearson Correlation | -.083 | -.128 | -.159 | -.197 | .411 |  |
|  | Sig. (2-tailed) | .570 | .381 | .275 | .199 | .491 |  |
|  | N | 49 | 49 | 49 | 44 | 5 |  |
| Channel_11  deoxy | Pearson Correlation | .117 | .160 | .144 | .197 | -.311 |  |
|  | Sig. (2-tailed) | .420 | .268 | .318 | .195 | .610 |  |
|  | N | 50 | 50 | 50 | 45 | 5 |  |
| Channel_12  deoxy | Pearson Correlation | .146 | ,300^*^ | .124 | .105 | .526 |  |
|  | Sig. (2-tailed) | .318 | .036 | .394 | .499 | .363 |  |
|  | N | 49 | 49 | 49 | 44 | 5 |  |
| Channel_13  deoxy | Pearson Correlation | .134 | .208 | .018 | .012 | -.350 |  |
|  | Sig. (2-tailed) | .353 | .147 | .901 | .938 | .564 |  |
|  | N | 50 | 50 | 50 | 45 | 5 |  |
| Channel_14  deoxy | Pearson Correlation | -.068 | .045 | .085 | -.008 | -.868 |  |
|  | Sig. (2-tailed) | .644 | .761 | .567 | .961 | .056 |  |
|  | N | 48 | 48 | 48 | 44 | 5 |  |
| Channel_15  deoxy | Pearson Correlation | .115 | .128 | -.073 | .003 | -.757 |  |
|  | Sig. (2-tailed) | .424 | .376 | .613 | .984 | .138 |  |
|  | N | 50 | 50 | 50 | 45 | 5 |  |
| Channel_16  deoxy | Pearson Correlation | -.119 | -.086 | -.030 | -.011 | .304 |  |
|  | Sig. (2-tailed) | .415 | .555 | .840 | .942 | .619 |  |
|  | N | 49 | 49 | 49 | 44 | 5 |  |
| Channel_17  deoxy | Pearson Correlation | .095 | .227 | .149 | .038 | .267 |  |
|  | Sig. (2-tailed) | .516 | .117 | .307 | .805 | .664 |  |
|  | N | 49 | 49 | 49 | 44 | 5 |  |
| Channel_18  deoxy | Pearson Correlation | .066 | .074 | .058 | .042 | -.643 |  |
|  | Sig. (2-tailed) | .646 | .605 | .684 | .783 | .242 |  |
|  | N | 51 | 51 | 51 | 46 | 5 |  |
| Channel_19  deoxy | Pearson Correlation | -.034 | .067 | -.041 | -.092 | .120 |  |
|  | Sig. (2-tailed) | .819 | .645 | .779 | .552 | .847 |  |
|  | N | 49 | 49 | 49 | 44 | 5 |  |
| Channel_20  deoxy | Pearson Correlation | -.148 | -.168 | -.166 | -.052 | -.539 |  |
|  | Sig. (2-tailed) | .303 | .244 | .250 | .735 | .348 |  |
|  | N | 50 | 50 | 50 | 45 | 5 |  |

*. Correlation is significant at the 0.05 level (2-tailed).

**. Correlation is significant at the 0.01 level (2-tailed).
