# Supplementary material for: Mutual interaction between motor cortex activation and pain in fibromyalgia: EEG-fNIRS study
Source: PLoS One. 2020 Jan 23;15(1):e0228158. doi: 10.1371/journal.pone.0228158 (PMC6977766; doi:10.1371/journal.pone.0228158)
Supplement: S6 Table — (DOCX) [file pone.0228158.s006.docx]

**S6 Table. Correlations for SFT condition.**

| **Correlations in SFT** | | | | | | |
| --- | --- | --- | --- | --- | --- | --- |
|  |  | Clinical Variable | | | | |
|  |  | sas | sds | maf | Disease Duration  (years) | wPi |
| Channel_1 | Pearson Correlation | -.139 | -.141 | -.225 | .092 | -.215 |
|  | Sig. (2-tailed) | .330 | .325 | .113 | .545 | .728 |
|  | N | 51 | 51 | 51 | 46 | 5 |
| Channel_2 | Pearson Correlation | -.245 | -.250 | -.194 | -.001 | -.520 |
|  | Sig. (2-tailed) | .084 | .076 | .173 | .995 | .369 |
|  | N | 51 | 51 | 51 | 46 | 5 |
| Channel_3 | Pearson Correlation | -.224 | -.255 | -.134 | .003 | -.381 |
|  | Sig. (2-tailed) | .118 | .073 | .355 | .982 | .527 |
|  | N | 50 | 50 | 50 | 45 | 5 |
| Channel_4 | Pearson Correlation | -.236 | -,287^*^ | -.275 | -.026 | -.165 |
|  | Sig. (2-tailed) | .095 | .041 | .051 | .865 | .791 |
|  | N | 51 | 51 | 51 | 46 | 5 |
| Channel_5 | Pearson Correlation | -.147 | -.140 | -.042 | .038 | -.049 |
|  | Sig. (2-tailed) | .302 | .327 | .769 | .802 | .938 |
|  | N | 51 | 51 | 51 | 46 | 5 |
| Channel_6 | Pearson Correlation | -.175 | -.188 | -.068 | -.088 | .168 |
|  | Sig. (2-tailed) | .219 | .187 | .638 | .560 | .787 |
|  | N | 51 | 51 | 51 | 46 | 5 |
| Channel_7 | Pearson Correlation | -.098 | -.149 | .029 | .077 | -.604 |
|  | Sig. (2-tailed) | .497 | .302 | .843 | .616 | .280 |
|  | N | 50 | 50 | 50 | 45 | 5 |
| Channel_8 | Pearson Correlation | -.004 | -.052 | .023 | .043 | -.252 |
|  | Sig. (2-tailed) | .975 | .715 | .872 | .777 | .682 |
|  | N | 51 | 51 | 51 | 46 | 5 |
| Channel_9 | Pearson Correlation | .169 | .077 | .060 | .181 | .073 |
|  | Sig. (2-tailed) | .241 | .597 | .680 | .235 | .907 |
|  | N | 50 | 50 | 50 | 45 | 5 |
| Channel_10 | Pearson Correlation | -.013 | -.209 | -.081 | -.003 | .461 |
|  | Sig. (2-tailed) | .927 | .150 | .580 | .984 | .435 |
|  | N | 49 | 49 | 49 | 44 | 5 |
| Channel_11 | Pearson Correlation | -.176 | -.099 | -.149 | .054 | -.667 |
|  | Sig. (2-tailed) | .221 | .495 | .302 | .725 | .219 |
|  | N | 50 | 50 | 50 | 45 | 5 |
| Channel_12 | Pearson Correlation | -.195 | -.120 | -.143 | .081 | -.877 |
|  | Sig. (2-tailed) | .180 | .412 | .328 | .601 | .051 |
|  | N | 49 | 49 | 49 | 44 | 5 |
| Channel_13 | Pearson Correlation | -.003 | -.100 | -.151 | .081 | -.772 |
|  | Sig. (2-tailed) | .985 | .490 | .294 | .597 | .126 |
|  | N | 50 | 50 | 50 | 45 | 5 |
| Channel_14 | Pearson Correlation | -.243 | -.190 | -.212 | .023 | -.723 |
|  | Sig. (2-tailed) | .095 | .195 | .148 | .883 | .167 |
|  | N | 48 | 48 | 48 | 44 | 5 |
| Channel_15 | Pearson Correlation | -.072 | -.174 | -.216 | -.085 | -.747 |
|  | Sig. (2-tailed) | .621 | .226 | .132 | .580 | .147 |
|  | N | 50 | 50 | 50 | 45 | 5 |
| Channel_16 | Pearson Correlation | -.118 | -.095 | -.025 | -.060 | .541 |
|  | Sig. (2-tailed) | .419 | .517 | .867 | .698 | .347 |
|  | N | 49 | 49 | 49 | 44 | 5 |
| Channel_17 | Pearson Correlation | -.071 | -.107 | .000 | .030 | -.794 |
|  | Sig. (2-tailed) | .626 | .463 | 1.000 | .846 | .109 |
|  | N | 49 | 49 | 49 | 44 | 5 |
| Channel_18 | Pearson Correlation | .034 | -.088 | -.156 | .004 | -.581 |
|  | Sig. (2-tailed) | .810 | .539 | .275 | .978 | .305 |
|  | N | 51 | 51 | 51 | 46 | 5 |
| Channel_19 | Pearson Correlation | -.026 | -.115 | -.167 | -.003 | -.489 |
|  | Sig. (2-tailed) | .861 | .432 | .251 | .982 | .403 |
|  | N | 49 | 49 | 49 | 44 | 5 |
| Channel_20 | Pearson Correlation | -.058 | -.090 | -.221 | -.116 | -.576 |
|  | Sig. (2-tailed) | .690 | .533 | .123 | .450 | .309 |
|  | N | 50 | 50 | 50 | 45 | 5 |
| Channel_1  deoxy | Pearson Correlation | .147 | .053 | ,316^*^ | -.251 | .147 |
|  | Sig. (2-tailed) | .303 | .710 | .027 | .684 | .303 |
|  | N | 51 | 51 | 49 | 5 | 51 |
| Channel_2  deoxy | Pearson Correlation | .243 | .168 | ,304^*^ | .443 | .243 |
|  | Sig. (2-tailed) | .086 | .240 | .033 | .455 | .086 |
|  | N | 51 | 51 | 49 | 5 | 51 |
| Channel_3  deoxy | Pearson Correlation | -.107 | -,387^**^ | -.102 | -.647 | -.107 |
|  | Sig. (2-tailed) | .459 | .006 | .490 | .238 | .459 |
|  | N | 50 | 50 | 48 | 5 | 50 |
| Channel_4  deoxy | Pearson Correlation | .129 | -.015 | .091 | .085 | .129 |
|  | Sig. (2-tailed) | .368 | .919 | .535 | .892 | .368 |
|  | N | 51 | 51 | 49 | 5 | 51 |
| Channel_5  deoxy | Pearson Correlation | -.069 | -,300^*^ | -.087 | ,880^*^ | -.069 |
|  | Sig. (2-tailed) | .631 | .032 | .551 | .049 | .631 |
|  | N | 51 | 51 | 49 | 5 | 51 |
| Channel_6  deoxy | Pearson Correlation | -,298^*^ | -,302^*^ | -,305^*^ | .311 | -,298^*^ |
|  | Sig. (2-tailed) | .034 | .031 | .033 | .610 | .034 |
|  | N | 51 | 51 | 49 | 5 | 51 |
| Channel_7  deoxy | Pearson Correlation | .140 | -.085 | .223 | .316 | .140 |
|  | Sig. (2-tailed) | .333 | .557 | .127 | .605 | .333 |
|  | N | 50 | 50 | 48 | 5 | 50 |
| Channel_8  deoxy | Pearson Correlation | .007 | -.111 | .086 | .231 | .007 |
|  | Sig. (2-tailed) | .960 | .440 | .558 | .708 | .960 |
|  | N | 51 | 51 | 49 | 5 | 51 |
| Channel_9  deoxy | Pearson Correlation | .132 | -.043 | -.035 | -.020 | .132 |
|  | Sig. (2-tailed) | .361 | .764 | .814 | .974 | .361 |
|  | N | 50 | 50 | 48 | 5 | 50 |
| Channel_10  deoxy | Pearson Correlation | .117 | -.032 | .071 | .477 | .117 |
|  | Sig. (2-tailed) | .425 | .827 | .637 | .417 | .425 |
|  | N | 49 | 49 | 47 | 5 | 49 |
| Channel_11  deoxy | Pearson Correlation | .183 | .041 | .167 | .229 | .183 |
|  | Sig. (2-tailed) | .202 | .775 | .258 | .711 | .202 |
|  | N | 50 | 50 | 48 | 5 | 50 |
| Channel_12  deoxy | Pearson Correlation | .127 | -.037 | .048 | ,920^*^ | .127 |
|  | Sig. (2-tailed) | .385 | .803 | .749 | .027 | .385 |
|  | N | 49 | 49 | 47 | 5 | 49 |
| Channel_13  deoxy | Pearson Correlation | .211 | -.002 | .055 | -.166 | .211 |
|  | Sig. (2-tailed) | .140 | .991 | .708 | .789 | .140 |
|  | N | 50 | 50 | 48 | 5 | 50 |
| Channel_14  deoxy | Pearson Correlation | -.053 | -.078 | .002 | -.723 | -.053 |
|  | Sig. (2-tailed) | .719 | .597 | .992 | .168 | .719 |
|  | N | 48 | 48 | 46 | 5 | 48 |
| Channel_15  deoxy | Pearson Correlation | .089 | -.186 | -.043 | -,910^*^ | .089 |
|  | Sig. (2-tailed) | .538 | .195 | .773 | .032 | .538 |
|  | N | 50 | 50 | 48 | 5 | 50 |
| Channel_16  deoxy | Pearson Correlation | -.085 | -.010 | .155 | .571 | -.085 |
|  | Sig. (2-tailed) | .560 | .943 | .297 | .315 | .560 |
|  | N | 49 | 49 | 47 | 5 | 49 |
| Channel_17  deoxy | Pearson Correlation | .125 | .073 | .156 | .846 | .125 |
|  | Sig. (2-tailed) | .392 | .618 | .294 | .071 | .392 |
|  | N | 49 | 49 | 47 | 5 | 49 |
| Channel_18  deoxy | Pearson Correlation | .039 | -.074 | .106 | .013 | .039 |
|  | Sig. (2-tailed) | .786 | .604 | .470 | .984 | .786 |
|  | N | 51 | 51 | 49 | 5 | 51 |
| Channel_19  deoxy | Pearson Correlation | .255 | -.077 | .014 | .762 | .255 |
|  | Sig. (2-tailed) | .077 | .599 | .927 | .135 | .077 |
|  | N | 49 | 49 | 47 | 5 | 49 |
| Channel_20  deoxy | Pearson Correlation | -.101 | -.182 | -.062 | -,881^*^ | -.101 |
|  | Sig. (2-tailed) | .485 | .205 | .678 | .048 | .485 |
|  | N | 50 | 50 | 48 | 5 | 50 |

*. Correlation is significant at the 0.05 level (2-tailed).

**. Correlation is significant at the 0.01 level (2-tailed).
