# Supplementary material for: Mutual interaction between motor cortex activation and pain in fibromyalgia: EEG-fNIRS study
Source: PLoS One. 2020 Jan 23;15(1):e0228158. doi: 10.1371/journal.pone.0228158 (PMC6977766; doi:10.1371/journal.pone.0228158)
Supplement: S7 Table — (DOCX) [file pone.0228158.s007.docx]

**S7 Table. Correlations for SFT + LASER ON THE LEFT HAND condition.**

| **Correlations in SFT + LASER ON THE LEFT HAND** | | | | | | |
| --- | --- | --- | --- | --- | --- | --- |
|  |  | Clinical Variable | | | | |
|  |  | sas | sds | maf | Disease Duration  (years) | wPi |
| Channel_1 | Pearson Correlation | -.245 | -.205 | -.024 | .047 | -.266 |
|  | Sig. (2-tailed) | .083 | .148 | .867 | .756 | .665 |
|  | N | 51 | 51 | 51 | 46 | 5 |
| Channel_2 | Pearson Correlation | -.240 | -.208 | .050 | .016 | -.152 |
|  | Sig. (2-tailed) | .090 | .143 | .728 | .915 | .807 |
|  | N | 51 | 51 | 51 | 46 | 5 |
| Channel_3 | Pearson Correlation | -.173 | -.156 | .139 | .013 | .492 |
|  | Sig. (2-tailed) | .231 | .278 | .334 | .934 | .400 |
|  | N | 50 | 50 | 50 | 45 | 5 |
| Channel_4 | Pearson Correlation | -.206 | -.199 | .021 | -.012 | -.828 |
|  | Sig. (2-tailed) | .146 | .163 | .883 | .935 | .083 |
|  | N | 51 | 51 | 51 | 46 | 5 |
| Channel_5 | Pearson Correlation | -.189 | -.123 | .163 | -.020 | -.722 |
|  | Sig. (2-tailed) | .184 | .391 | .254 | .894 | .169 |
|  | N | 51 | 51 | 51 | 46 | 5 |
| Channel_6 | Pearson Correlation | -.211 | -.200 | .009 | -.137 | -.763 |
|  | Sig. (2-tailed) | .138 | .159 | .951 | .364 | .134 |
|  | N | 51 | 51 | 51 | 46 | 5 |
| Channel_7 | Pearson Correlation | -.118 | -.122 | .182 | .054 | -.736 |
|  | Sig. (2-tailed) | .416 | .400 | .205 | .724 | .157 |
|  | N | 50 | 50 | 50 | 45 | 5 |
| Channel_8 | Pearson Correlation | -.024 | -.028 | .206 | .036 | .047 |
|  | Sig. (2-tailed) | .865 | .846 | .148 | .814 | .940 |
|  | N | 51 | 51 | 51 | 46 | 5 |
| Channel_9 | Pearson Correlation | .201 | .115 | .242 | .184 | ,880^*^ |
|  | Sig. (2-tailed) | .163 | .427 | .091 | .226 | .049 |
|  | N | 50 | 50 | 50 | 45 | 5 |
| Channel_10 | Pearson Correlation | .025 | -.105 | .026 | -.150 | -.035 |
|  | Sig. (2-tailed) | .864 | .473 | .858 | .332 | .955 |
|  | N | 49 | 49 | 49 | 44 | 5 |
| Channel_11 | Pearson Correlation | -.124 | -.078 | .070 | .046 | -.163 |
|  | Sig. (2-tailed) | .390 | .591 | .631 | .764 | .793 |
|  | N | 50 | 50 | 50 | 45 | 5 |
| Channel_12 | Pearson Correlation | -.104 | -.070 | .068 | .078 | -.316 |
|  | Sig. (2-tailed) | .475 | .634 | .641 | .613 | .604 |
|  | N | 49 | 49 | 49 | 44 | 5 |
| Channel_13 | Pearson Correlation | .066 | .012 | .120 | .034 | -.436 |
|  | Sig. (2-tailed) | .647 | .936 | .408 | .823 | .463 |
|  | N | 50 | 50 | 50 | 45 | 5 |
| Channel_14 | Pearson Correlation | -.267 | -.196 | -.002 | -.078 | .359 |
|  | Sig. (2-tailed) | .066 | .182 | .987 | .616 | .553 |
|  | N | 48 | 48 | 48 | 44 | 5 |
| Channel_15 | Pearson Correlation | .062 | .002 | .059 | -.018 | .433 |
|  | Sig. (2-tailed) | .669 | .988 | .683 | .907 | .467 |
|  | N | 50 | 50 | 50 | 45 | 5 |
| Channel_16 | Pearson Correlation | -.116 | -.091 | -.003 | -.190 | .415 |
|  | Sig. (2-tailed) | .429 | .536 | .982 | .218 | .487 |
|  | N | 49 | 49 | 49 | 44 | 5 |
| Channel_17 | Pearson Correlation | -.046 | -.070 | .128 | -.023 | -.537 |
|  | Sig. (2-tailed) | .754 | .630 | .382 | .881 | .351 |
|  | N | 49 | 49 | 49 | 44 | 5 |
| Channel_18 | Pearson Correlation | -.057 | -.179 | -.015 | -.125 | -.589 |
|  | Sig. (2-tailed) | .691 | .210 | .919 | .407 | .296 |
|  | N | 51 | 51 | 51 | 46 | 5 |
| Channel_19 | Pearson Correlation | -.143 | -.204 | -.092 | -.167 | -.387 |
|  | Sig. (2-tailed) | .328 | .160 | .528 | .278 | .520 |
|  | N | 49 | 49 | 49 | 44 | 5 |
| Channel_20 | Pearson Correlation | -.052 | -.076 | -.124 | -.223 | -.578 |
|  | Sig. (2-tailed) | .720 | .601 | .391 | .141 | .308 |
|  | N | 50 | 50 | 50 | 45 | 5 |
| Channel_1  deoxy | Pearson Correlation | -.184 | -.079 | .069 | -.072 | -.155 |
|  | Sig. (2-tailed) | .195 | .583 | .629 | .636 | .803 |
|  | N | 51 | 51 | 51 | 46 | 5 |
| Channel_2  deoxy | Pearson Correlation | .032 | .178 | .207 | -.049 | -.228 |
|  | Sig. (2-tailed) | .823 | .212 | .146 | .747 | .712 |
|  | N | 51 | 51 | 51 | 46 | 5 |
| Channel_3  deoxy | Pearson Correlation | -,319^*^ | -.201 | -.176 | -.178 | .386 |
|  | Sig. (2-tailed) | .024 | .162 | .222 | .242 | .522 |
|  | N | 50 | 50 | 50 | 45 | 5 |
| Channel_4  deoxy | Pearson Correlation | .031 | .137 | .057 | -.008 | .081 |
|  | Sig. (2-tailed) | .830 | .337 | .690 | .956 | .897 |
|  | N | 51 | 51 | 51 | 46 | 5 |
| Channel_5  deoxy | Pearson Correlation | -,379^**^ | -.181 | -.219 | -.093 | -.754 |
|  | Sig. (2-tailed) | .006 | .205 | .123 | .540 | .141 |
|  | N | 51 | 51 | 51 | 46 | 5 |
| Channel_6  deoxy | Pearson Correlation | -,278^*^ | -,281^*^ | -.218 | -.172 | .236 |
|  | Sig. (2-tailed) | .048 | .046 | .124 | .252 | .703 |
|  | N | 51 | 51 | 51 | 46 | 5 |
| Channel_7  deoxy | Pearson Correlation | .099 | .208 | .138 | .009 | .454 |
|  | Sig. (2-tailed) | .494 | .147 | .340 | .953 | .442 |
|  | N | 50 | 50 | 50 | 45 | 5 |
| Channel_8  deoxy | Pearson Correlation | -.067 | -.020 | .010 | -.142 | .083 |
|  | Sig. (2-tailed) | .639 | .888 | .942 | .346 | .895 |
|  | N | 51 | 51 | 51 | 46 | 5 |
| Channel_9  deoxy | Pearson Correlation | -,302^*^ | -.222 | -.071 | -.229 | -.037 |
|  | Sig. (2-tailed) | .033 | .122 | .624 | .131 | .953 |
|  | N | 50 | 50 | 50 | 45 | 5 |
| Channel_10  deoxy | Pearson Correlation | -.087 | -.066 | -.152 | -.154 | -.377 |
|  | Sig. (2-tailed) | .550 | .650 | .296 | .317 | .532 |
|  | N | 49 | 49 | 49 | 44 | 5 |
| Channel_11  deoxy | Pearson Correlation | -.062 | .096 | .035 | .054 | .378 |
|  | Sig. (2-tailed) | .668 | .508 | .808 | .727 | .530 |
|  | N | 50 | 50 | 50 | 45 | 5 |
| Channel_12  deoxy | Pearson Correlation | .138 | .231 | .218 | .054 | .618 |
|  | Sig. (2-tailed) | .345 | .110 | .133 | .729 | .266 |
|  | N | 49 | 49 | 49 | 44 | 5 |
| Channel_13  deoxy | Pearson Correlation | .128 | .172 | .081 | .004 | .057 |
|  | Sig. (2-tailed) | .377 | .233 | .574 | .978 | .928 |
|  | N | 50 | 50 | 50 | 45 | 5 |
| Channel_14  deoxy | Pearson Correlation | -.272 | -.111 | -.005 | -.210 | -.021 |
|  | Sig. (2-tailed) | .062 | .453 | .973 | .172 | .974 |
|  | N | 48 | 48 | 48 | 44 | 5 |
| Channel_15  deoxy | Pearson Correlation | .171 | .174 | -.015 | .066 | -.407 |
|  | Sig. (2-tailed) | .236 | .227 | .916 | .667 | .497 |
|  | N | 50 | 50 | 50 | 45 | 5 |
| Channel_16  deoxy | Pearson Correlation | -.117 | -.087 | -.016 | -.098 | -.429 |
|  | Sig. (2-tailed) | .425 | .550 | .912 | .525 | .471 |
|  | N | 49 | 49 | 49 | 44 | 5 |
| Channel_17  deoxy | Pearson Correlation | .098 | .135 | .231 | -.032 | .468 |
|  | Sig. (2-tailed) | .501 | .353 | .111 | .837 | .426 |
|  | N | 49 | 49 | 49 | 44 | 5 |
| Channel_18  deoxy | Pearson Correlation | .112 | .092 | .070 | -.137 | -.491 |
|  | Sig. (2-tailed) | .435 | .520 | .623 | .363 | .401 |
|  | N | 51 | 51 | 51 | 46 | 5 |
| Channel_19  deoxy | Pearson Correlation | .065 | .162 | -.029 | -.236 | .123 |
|  | Sig. (2-tailed) | .659 | .267 | .845 | .123 | .843 |
|  | N | 49 | 49 | 49 | 44 | 5 |
| Channel_20  deoxy | Pearson Correlation | -.051 | -.066 | -.100 | .003 | -.557 |
|  | Sig. (2-tailed) | .725 | .648 | .491 | .984 | .330 |
|  | N | 50 | 50 | 50 | 45 | 5 |

*. Correlation is significant at the 0.05 level (2-tailed).

**. Correlation is significant at the 0.01 level (2-tailed).
