# Supplementary material for: Mutual interaction between motor cortex activation and pain in fibromyalgia: EEG-fNIRS study
Source: PLoS One. 2020 Jan 23;15(1):e0228158. doi: 10.1371/journal.pone.0228158 (PMC6977766; doi:10.1371/journal.pone.0228158)
Supplement: S8 Table — (DOCX) [file pone.0228158.s008.docx]

**S8 Table. Correlations for SFT + LASER ON THE RIGHT HAND condition.**

| **Correlations in SFT + LASER ON THE RIGHT HAND** | | | | | | |
| --- | --- | --- | --- | --- | --- | --- |
|  |  | Clinical Variable | | | | |
|  |  | sas | sds | maf | Disease Duration  (years) | wPi |
| Channel_1 | Pearson Correlation | -.154 | -.090 | -.124 | .090 | -.544 |
|  | Sig. (2-tailed) | .281 | .532 | .386 | .553 | .343 |
|  | N | 51 | 51 | 51 | 46 | 5 |
| Channel_2 | Pearson Correlation | -.198 | -.195 | -.059 | .009 | -.597 |
|  | Sig. (2-tailed) | .163 | .170 | .683 | .952 | .288 |
|  | N | 51 | 51 | 51 | 46 | 5 |
| Channel_3 | Pearson Correlation | -.183 | -.225 | -.025 | -.014 | -.548 |
|  | Sig. (2-tailed) | .202 | .116 | .864 | .929 | .339 |
|  | N | 50 | 50 | 50 | 45 | 5 |
| Channel_4 | Pearson Correlation | -.215 | -.217 | -.103 | -.053 | -.585 |
|  | Sig. (2-tailed) | .129 | .125 | .473 | .729 | .300 |
|  | N | 51 | 51 | 51 | 46 | 5 |
| Channel_5 | Pearson Correlation | -.220 | -.231 | -.036 | -.070 | -.264 |
|  | Sig. (2-tailed) | .120 | .103 | .801 | .642 | .668 |
|  | N | 51 | 51 | 51 | 46 | 5 |
| Channel_6 | Pearson Correlation | -.219 | -.209 | -.079 | -.132 | -.168 |
|  | Sig. (2-tailed) | .123 | .141 | .579 | .383 | .786 |
|  | N | 51 | 51 | 51 | 46 | 5 |
| Channel_7 | Pearson Correlation | -.104 | -.132 | .068 | .038 | -.654 |
|  | Sig. (2-tailed) | .473 | .361 | .639 | .803 | .231 |
|  | N | 50 | 50 | 50 | 45 | 5 |
| Channel_8 | Pearson Correlation | -.109 | -.177 | .026 | -.001 | -.607 |
|  | Sig. (2-tailed) | .448 | .215 | .858 | .996 | .278 |
|  | N | 51 | 51 | 51 | 46 | 5 |
| Channel_9 | Pearson Correlation | .123 | -.004 | .129 | .157 | .007 |
|  | Sig. (2-tailed) | .394 | .977 | .373 | .302 | .991 |
|  | N | 50 | 50 | 50 | 45 | 5 |
| Channel_10 | Pearson Correlation | .012 | -.128 | -.032 | -.131 | .543 |
|  | Sig. (2-tailed) | .933 | .383 | .827 | .396 | .344 |
|  | N | 49 | 49 | 49 | 44 | 5 |
| Channel_11 | Pearson Correlation | -.189 | -.119 | -.026 | .034 | -.591 |
|  | Sig. (2-tailed) | .189 | .410 | .860 | .823 | .294 |
|  | N | 50 | 50 | 50 | 45 | 5 |
| Channel_12 | Pearson Correlation | -.198 | -.173 | -.057 | .082 | -.459 |
|  | Sig. (2-tailed) | .174 | .235 | .695 | .597 | .437 |
|  | N | 49 | 49 | 49 | 44 | 5 |
| Channel_13 | Pearson Correlation | -.015 | -.110 | -.029 | .070 | -.540 |
|  | Sig. (2-tailed) | .916 | .448 | .843 | .648 | .348 |
|  | N | 50 | 50 | 50 | 45 | 5 |
| Channel_14 | Pearson Correlation | -,295^*^ | -.266 | -.129 | -.051 | -.846 |
|  | Sig. (2-tailed) | .042 | .067 | .382 | .740 | .071 |
|  | N | 48 | 48 | 48 | 44 | 5 |
| Channel_15 | Pearson Correlation | -.078 | -.141 | -.079 | -.060 | -.486 |
|  | Sig. (2-tailed) | .589 | .327 | .584 | .695 | .407 |
|  | N | 50 | 50 | 50 | 45 | 5 |
| Channel_16 | Pearson Correlation | -.123 | -.099 | -.023 | -.149 | .451 |
|  | Sig. (2-tailed) | .398 | .500 | .875 | .334 | .446 |
|  | N | 49 | 49 | 49 | 44 | 5 |
| Channel_17 | Pearson Correlation | -.093 | -.160 | .037 | -.005 | -.497 |
|  | Sig. (2-tailed) | .525 | .272 | .803 | .977 | .394 |
|  | N | 49 | 49 | 49 | 44 | 5 |
| Channel_18 | Pearson Correlation | -.037 | -.166 | -.106 | .003 | -.427 |
|  | Sig. (2-tailed) | .794 | .245 | .460 | .982 | .474 |
|  | N | 51 | 51 | 51 | 46 | 5 |
| Channel_19 | Pearson Correlation | -.234 | -,299^*^ | -.278 | -.099 | -.429 |
|  | Sig. (2-tailed) | .105 | .037 | .053 | .522 | .471 |
|  | N | 49 | 49 | 49 | 44 | 5 |
| Channel_20 | Pearson Correlation | -.066 | -.090 | -.143 | -.190 | -.567 |
|  | Sig. (2-tailed) | .650 | .533 | .322 | .212 | .319 |
|  | N | 50 | 50 | 50 | 45 | 5 |
| Channel_1  deoxy | Pearson Correlation | .019 | .200 | .143 | .171 | -.495 |
|  | Sig. (2-tailed) | .894 | .159 | .318 | .255 | .397 |
|  | N | 51 | 51 | 51 | 46 | 5 |
| Channel_2  deoxy | Pearson Correlation | .133 | .242 | .237 | .069 | -.432 |
|  | Sig. (2-tailed) | .350 | .087 | .094 | .646 | .467 |
|  | N | 51 | 51 | 51 | 46 | 5 |
| Channel_3  deoxy | Pearson Correlation | -.218 | -.131 | -.262 | -.027 | -.614 |
|  | Sig. (2-tailed) | .129 | .366 | .066 | .859 | .270 |
|  | N | 50 | 50 | 50 | 45 | 5 |
| Channel_4  deoxy | Pearson Correlation | .079 | .187 | .076 | .123 | .213 |
|  | Sig. (2-tailed) | .582 | .189 | .596 | .415 | .730 |
|  | N | 51 | 51 | 51 | 46 | 5 |
| Channel_5  deoxy | Pearson Correlation | -.200 | -.036 | -.190 | .010 | .368 |
|  | Sig. (2-tailed) | .160 | .801 | .183 | .950 | .542 |
|  | N | 51 | 51 | 51 | 46 | 5 |
| Channel_6  deoxy | Pearson Correlation | -,318^*^ | -,319^*^ | -,315^*^ | -.148 | .443 |
|  | Sig. (2-tailed) | .023 | .023 | .024 | .326 | .455 |
|  | N | 51 | 51 | 51 | 46 | 5 |
| Channel_7  deoxy | Pearson Correlation | .197 | .259 | .094 | .280 | -.378 |
|  | Sig. (2-tailed) | .170 | .070 | .516 | .062 | .530 |
|  | N | 50 | 50 | 50 | 45 | 5 |
| Channel_8  deoxy | Pearson Correlation | -.098 | -.089 | -.118 | -.031 | -.188 |
|  | Sig. (2-tailed) | .494 | .533 | .411 | .839 | .762 |
|  | N | 51 | 51 | 51 | 46 | 5 |
| Channel_9  deoxy | Pearson Correlation | -.118 | -.104 | -.128 | -.074 | -.279 |
|  | Sig. (2-tailed) | .414 | .472 | .376 | .628 | .649 |
|  | N | 50 | 50 | 50 | 45 | 5 |
| Channel_10  deoxy | Pearson Correlation | -.198 | -.200 | -.266 | -.218 | .819 |
|  | Sig. (2-tailed) | .174 | .169 | .065 | .155 | .090 |
|  | N | 49 | 49 | 49 | 44 | 5 |
| Channel_11  deoxy | Pearson Correlation | .038 | .164 | .135 | .184 | -.261 |
|  | Sig. (2-tailed) | .791 | .255 | .349 | .226 | .671 |
|  | N | 50 | 50 | 50 | 45 | 5 |
| Channel_12  deoxy | Pearson Correlation | .180 | .217 | .102 | .122 | .652 |
|  | Sig. (2-tailed) | .216 | .135 | .486 | .431 | .233 |
|  | N | 49 | 49 | 49 | 44 | 5 |
| Channel_13  deoxy | Pearson Correlation | .149 | .178 | .065 | .066 | -.555 |
|  | Sig. (2-tailed) | .303 | .217 | .654 | .668 | .332 |
|  | N | 50 | 50 | 50 | 45 | 5 |
| Channel_14  deoxy | Pearson Correlation | -.064 | .032 | .016 | .050 | -.724 |
|  | Sig. (2-tailed) | .666 | .827 | .913 | .749 | .167 |
|  | N | 48 | 48 | 48 | 44 | 5 |
| Channel_15  deoxy | Pearson Correlation | .201 | .222 | -.017 | .081 | -.457 |
|  | Sig. (2-tailed) | .162 | .122 | .905 | .596 | .439 |
|  | N | 50 | 50 | 50 | 45 | 5 |
| Channel_16  deoxy | Pearson Correlation | -.113 | -.083 | -.010 | .104 | -.513 |
|  | Sig. (2-tailed) | .439 | .569 | .946 | .502 | .377 |
|  | N | 49 | 49 | 49 | 44 | 5 |
| Channel_17  deoxy | Pearson Correlation | .141 | .113 | .147 | .107 | .467 |
|  | Sig. (2-tailed) | .334 | .439 | .313 | .489 | .428 |
|  | N | 49 | 49 | 49 | 44 | 5 |
| Channel_18  deoxy | Pearson Correlation | .166 | .060 | .033 | .077 | -.811 |
|  | Sig. (2-tailed) | .244 | .677 | .818 | .610 | .096 |
|  | N | 51 | 51 | 51 | 46 | 5 |
| Channel_19  deoxy | Pearson Correlation | .078 | .158 | -.119 | -.037 | .021 |
|  | Sig. (2-tailed) | .595 | .278 | .414 | .813 | .974 |
|  | N | 49 | 49 | 49 | 44 | 5 |
| Channel_20  deoxy | Pearson Correlation | -.057 | -.077 | -.123 | .017 | -.557 |
|  | Sig. (2-tailed) | .696 | .593 | .394 | .910 | .329 |
|  | N | 50 | 50 | 50 | 45 | 5 |

*. Correlation is significant at the 0.05 level (2-tailed).

**. Correlation is significant at the 0.01 level (2-tailed).
