# Supplementary material for: Mutual interaction between motor cortex activation and pain in fibromyalgia: EEG-fNIRS study
Source: PLoS One. 2020 Jan 23;15(1):e0228158. doi: 10.1371/journal.pone.0228158 (PMC6977766; doi:10.1371/journal.pone.0228158)
Supplement: S9 Table — (DOCX) [file pone.0228158.s009.docx]

**S9 Table. Correlations for baseline condition**

| **Correlations in BASELINE** | | | | | | |
| --- | --- | --- | --- | --- | --- | --- |
|  |  | Clinical Variable | | | | |
|  |  | sas | sds | maf | Disease Duration  (years) | wPi |
| Channel_1 | Pearson Correlation | -.083 | -.090 | -.103 | .140 | .358 |
|  | Sig. (2-tailed) | .562 | .531 | .471 | .352 | .554 |
|  | N | 51 | 51 | 51 | 46 | 5 |
| Channel_2 | Pearson Correlation | -.171 | -.151 | -.084 | .045 | .335 |
|  | Sig. (2-tailed) | .229 | .291 | .556 | .768 | .582 |
|  | N | 51 | 51 | 51 | 46 | 5 |
| Channel_3 | Pearson Correlation | -.117 | -.126 | .006 | .029 | ,899^*^ |
|  | Sig. (2-tailed) | .417 | .382 | .969 | .852 | .038 |
|  | N | 50 | 50 | 50 | 45 | 5 |
| Channel_4 | Pearson Correlation | -.126 | -.135 | -.046 | -.038 | -.363 |
|  | Sig. (2-tailed) | .377 | .344 | .747 | .803 | .548 |
|  | N | 51 | 51 | 51 | 46 | 5 |
| Channel_5 | Pearson Correlation | -.197 | -.145 | .043 | -.099 | .445 |
|  | Sig. (2-tailed) | .165 | .310 | .764 | .513 | .452 |
|  | N | 51 | 51 | 51 | 46 | 5 |
| Channel_6 | Pearson Correlation | -.226 | -.193 | -.091 | -.165 | .087 |
|  | Sig. (2-tailed) | .111 | .175 | .524 | .273 | .889 |
|  | N | 51 | 51 | 51 | 46 | 5 |
| Channel_7 | Pearson Correlation | -.039 | -.078 | .079 | .053 | -.314 |
|  | Sig. (2-tailed) | .789 | .591 | .586 | .729 | .607 |
|  | N | 50 | 50 | 50 | 45 | 5 |
| Channel_8 | Pearson Correlation | -.074 | -.070 | -.010 | -.012 | -.035 |
|  | Sig. (2-tailed) | .607 | .624 | .944 | .937 | .955 |
|  | N | 51 | 51 | 51 | 46 | 5 |
| Channel_9 | Pearson Correlation | .175 | .090 | .128 | .158 | .562 |
|  | Sig. (2-tailed) | .224 | .533 | .375 | .300 | .324 |
|  | N | 50 | 50 | 50 | 45 | 5 |
| Channel_10 | Pearson Correlation | -.044 | -.178 | -.058 | -.247 | .633 |
|  | Sig. (2-tailed) | .762 | .221 | .692 | .107 | .251 |
|  | N | 49 | 49 | 49 | 44 | 5 |
| Channel_11 | Pearson Correlation | -.197 | -.117 | -.069 | .106 | .122 |
|  | Sig. (2-tailed) | .170 | .418 | .633 | .489 | .845 |
|  | N | 50 | 50 | 50 | 45 | 5 |
| Channel_12 | Pearson Correlation | -.169 | -.120 | -.109 | .082 | -.140 |
|  | Sig. (2-tailed) | .246 | .412 | .456 | .596 | .822 |
|  | N | 49 | 49 | 49 | 44 | 5 |
| Channel_13 | Pearson Correlation | .008 | -.074 | -.080 | .072 | -.191 |
|  | Sig. (2-tailed) | .955 | .609 | .579 | .640 | .758 |
|  | N | 50 | 50 | 50 | 45 | 5 |
| Channel_14 | Pearson Correlation | -.276 | -.204 | -.083 | -.051 | .214 |
|  | Sig. (2-tailed) | .058 | .165 | .576 | .743 | .730 |
|  | N | 48 | 48 | 48 | 44 | 5 |
| Channel_15 | Pearson Correlation | -.100 | -.127 | -.102 | -.133 | .463 |
|  | Sig. (2-tailed) | .489 | .381 | .481 | .382 | .432 |
|  | N | 50 | 50 | 50 | 45 | 5 |
| Channel_16 | Pearson Correlation | -.124 | -.095 | -.034 | -.226 | .546 |
|  | Sig. (2-tailed) | .397 | .517 | .819 | .141 | .341 |
|  | N | 49 | 49 | 49 | 44 | 5 |
| Channel_17 | Pearson Correlation | -.138 | -.150 | -.007 | -.024 | -.664 |
|  | Sig. (2-tailed) | .346 | .303 | .961 | .876 | .222 |
|  | N | 49 | 49 | 49 | 44 | 5 |
| Channel_18 | Pearson Correlation | -.050 | -.137 | -.151 | -.060 | -.287 |
|  | Sig. (2-tailed) | .727 | .338 | .291 | .692 | .639 |
|  | N | 51 | 51 | 51 | 46 | 5 |
| Channel_19 | Pearson Correlation | -.163 | -.174 | -.232 | -.110 | -.214 |
|  | Sig. (2-tailed) | .263 | .231 | .108 | .479 | .730 |
|  | N | 49 | 49 | 49 | 44 | 5 |
| Channel_20 | Pearson Correlation | -.068 | -.093 | -.149 | -,319^*^ | -.566 |
|  | Sig. (2-tailed) | .639 | .522 | .301 | .033 | .320 |
|  | N | 50 | 50 | 50 | 45 | 5 |
| Channel_1  deoxy | Pearson Correlation | .054 | .144 | .176 | .174 | .069 |
|  | Sig. (2-tailed) | .706 | .312 | .217 | .247 | .913 |
|  | N | 51 | 51 | 51 | 46 | 5 |
| Channel_2  deoxy | Pearson Correlation | .140 | .224 | .259 | -.074 | .322 |
|  | Sig. (2-tailed) | .327 | .114 | .066 | .625 | .597 |
|  | N | 51 | 51 | 51 | 46 | 5 |
| Channel_3  deoxy | Pearson Correlation | -.196 | -.071 | -.200 | -.133 | .601 |
|  | Sig. (2-tailed) | .172 | .622 | .165 | .382 | .284 |
|  | N | 50 | 50 | 50 | 45 | 5 |
| Channel_4  deoxy | Pearson Correlation | .079 | .091 | .006 | -.006 | -.217 |
|  | Sig. (2-tailed) | .583 | .525 | .965 | .969 | .725 |
|  | N | 51 | 51 | 51 | 46 | 5 |
| Channel_5  deoxy | Pearson Correlation | -.240 | -.064 | -.231 | -.129 | -.690 |
|  | Sig. (2-tailed) | .090 | .656 | .102 | .395 | .197 |
|  | N | 51 | 51 | 51 | 46 | 5 |
| Channel_6  deoxy | Pearson Correlation | -.246 | -.157 | -,296^*^ | -.168 | .389 |
|  | Sig. (2-tailed) | .082 | .271 | .035 | .265 | .517 |
|  | N | 51 | 51 | 51 | 46 | 5 |
| Channel_7  deoxy | Pearson Correlation | .227 | .157 | .098 | .056 | .497 |
|  | Sig. (2-tailed) | .114 | .277 | .497 | .713 | .394 |
|  | N | 50 | 50 | 50 | 45 | 5 |
| Channel_8  deoxy | Pearson Correlation | -.059 | .003 | -.049 | -.164 | -.078 |
|  | Sig. (2-tailed) | .680 | .981 | .732 | .275 | .901 |
|  | N | 51 | 51 | 51 | 46 | 5 |
| Channel_9  deoxy | Pearson Correlation | -.084 | -.053 | -.091 | -.105 | -.350 |
|  | Sig. (2-tailed) | .564 | .714 | .530 | .492 | .564 |
|  | N | 50 | 50 | 50 | 45 | 5 |
| Channel_10  deoxy | Pearson Correlation | -.136 | -.132 | -.179 | -.205 | .636 |
|  | Sig. (2-tailed) | .350 | .367 | .217 | .183 | .249 |
|  | N | 49 | 49 | 49 | 44 | 5 |
| Channel_11  deoxy | Pearson Correlation | .029 | .072 | .198 | .112 | .660 |
|  | Sig. (2-tailed) | .840 | .618 | .168 | .464 | .226 |
|  | N | 50 | 50 | 50 | 45 | 5 |
| Channel_12  deoxy | Pearson Correlation | .060 | .121 | .089 | .019 | ,887^*^ |
|  | Sig. (2-tailed) | .684 | .406 | .542 | .901 | .045 |
|  | N | 49 | 49 | 49 | 44 | 5 |
| Channel_13  deoxy | Pearson Correlation | .081 | .042 | .065 | -.097 | .703 |
|  | Sig. (2-tailed) | .578 | .771 | .655 | .524 | .186 |
|  | N | 50 | 50 | 50 | 45 | 5 |
| Channel_14  deoxy | Pearson Correlation | -.100 | -.053 | .145 | -.128 | .074 |
|  | Sig. (2-tailed) | .500 | .721 | .325 | .409 | .906 |
|  | N | 48 | 48 | 48 | 44 | 5 |
| Channel_15  deoxy | Pearson Correlation | .059 | .024 | -.043 | -.163 | .278 |
|  | Sig. (2-tailed) | .685 | .870 | .765 | .285 | .651 |
|  | N | 50 | 50 | 50 | 45 | 5 |
| Channel_16  deoxy | Pearson Correlation | -.115 | -.087 | -.024 | .135 | -.677 |
|  | Sig. (2-tailed) | .433 | .550 | .868 | .382 | .209 |
|  | N | 49 | 49 | 49 | 44 | 5 |
| Channel_17  deoxy | Pearson Correlation | .089 | .094 | .178 | -.066 | .477 |
|  | Sig. (2-tailed) | .541 | .521 | .221 | .672 | .416 |
|  | N | 49 | 49 | 49 | 44 | 5 |
| Channel_18  deoxy | Pearson Correlation | .073 | -.019 | .026 | -.042 | .134 |
|  | Sig. (2-tailed) | .613 | .895 | .859 | .780 | .830 |
|  | N | 51 | 51 | 51 | 46 | 5 |
| Channel_19  deoxy | Pearson Correlation | .053 | .154 | -.061 | -.139 | .432 |
|  | Sig. (2-tailed) | .720 | .290 | .677 | .368 | .468 |
|  | N | 49 | 49 | 49 | 44 | 5 |
| Channel_20  deoxy | Pearson Correlation | -.065 | -.083 | -.120 | -.086 | -.552 |
|  | Sig. (2-tailed) | .652 | .568 | .408 | .574 | .335 |
|  | N | 50 | 50 | 50 | 45 | 5 |

*. Correlation is significant at the 0.05 level (2-tailed).

**. Correlation is significant at the 0.01 level (2-tailed).
