# Supplementary material for: Mutual interaction between motor cortex activation and pain in fibromyalgia: EEG-fNIRS study
Source: PLoS One. 2020 Jan 23;15(1):e0228158. doi: 10.1371/journal.pone.0228158 (PMC6977766; doi:10.1371/journal.pone.0228158)
Supplement: S10 Table — (DOCX) [file pone.0228158.s010.docx]

**S10 Table. Correlations for FFT condition.**

| **Correlations in FFT** | | | | | | |
| --- | --- | --- | --- | --- | --- | --- |
|  | **ΔHbO_2_** | | | **ΔHb** | | |
|  | SPEED | | | SPEED | | |
| Channel | Pearson Correlation | Sig.  (2-tailed) | N | Pearson Correlation | Sig.  (2-tailed) | N |
| Channel_1 | ,057 | ,673 | 58 | -,136 | ,308 | 58 |
| Channel_2 | ,037 | ,783 | 58 | -,103 | ,441 | 58 |
| Channel_3 | ,022 | ,873 | 57 | ,137 | ,311 | 57 |
| Channel_4 | ,062 | ,642 | 58 | -,068 | ,613 | 58 |
| Channel_5 | ,004 | ,979 | 58 | ,077 | ,568 | 58 |
| Channel_6 | ,253 | ,055 | 58 | ,043 | ,750 | 58 |
| Channel_7 | ,050 | ,712 | 57 | -,012 | ,928 | 57 |
| Channel_8 | -,003 | ,984 | 58 | -,042 | ,754 | 58 |
| Channel_9 | ,042 | ,754 | 57 | ,142 | ,293 | 57 |
| Channel_10 | ,073 | ,592 | 56 | ,136 | ,317 | 56 |
| Channel_11 | ,014 | ,917 | 57 | -,059 | ,661 | 57 |
| Channel_12 | ,082 | ,547 | 56 | ,046 | ,736 | 56 |
| Channel_13 | ,044 | ,747 | 56 | ,066 | ,630 | 56 |
| Channel_14 | -,052 | ,705 | 55 | -,055 | ,689 | 55 |
| Channel_15 | ,022 | ,870 | 57 | ,085 | ,529 | 57 |
| Channel_16 | ,006 | ,964 | 56 | -,020 | ,884 | 56 |
| Channel_17 | ,041 | ,763 | 56 | -,044 | ,749 | 56 |
| Channel_18 | ,134 | ,316 | 58 | ,123 | ,357 | 58 |
| Channel_19 | ,279^*^ | ,038 | 56 | ,225 | ,096 | 56 |
| Channel_20 | ,185 | ,172 | 56 | ,171 | ,209 | 56 |

*. Correlation is significant at the 0.05 level (2-tailed).

**. Correlation is significant at the 0.01 level (2-tailed).
