# Supplementary material for: Mutual interaction between motor cortex activation and pain in fibromyalgia: EEG-fNIRS study
Source: PLoS One. 2020 Jan 23;15(1):e0228158. doi: 10.1371/journal.pone.0228158 (PMC6977766; doi:10.1371/journal.pone.0228158)
Supplement: S11 Table — (DOCX) [file pone.0228158.s011.docx]

**S11 Table. Correlations for FFT + LASER ON THE RIGHT HAND condition.**

| **Correlations in FFT + LASER ON THE RIGHT HAND** | | | | | | |
| --- | --- | --- | --- | --- | --- | --- |
|  | **ΔHbO_2_** | | | **ΔHb** | | |
|  | SPEED | | | SPEED | | |
| Channel | Pearson Correlation | Sig.  (2-tailed) | N | Pearson Correlation | Sig.  (2-tailed) | N |
| Channel_1 | -,025 | ,854 | 58 | -,059 | ,658 | 58 |
| Channel_2 | ,005 | ,972 | 58 | ,056 | ,679 | 58 |
| Channel_3 | -,006 | ,967 | 57 | ,233 | ,081 | 57 |
| Channel_4 | ,061 | ,647 | 58 | ,047 | ,728 | 58 |
| Channel_5 | -,018 | ,895 | 58 | ,078 | ,560 | 58 |
| Channel_6 | ,169 | ,204 | 58 | ,072 | ,590 | 58 |
| Channel_7 | -,044 | ,748 | 57 | -,016 | ,906 | 57 |
| Channel_8 | -,073 | ,588 | 58 | ,072 | ,589 | 58 |
| Channel_9 | -,104 | ,440 | 57 | ,292 | ,028 | 57 |
| Channel_10 | ,103 | ,449 | 56 | ,155 | ,253 | 56 |
| Channel_11 | -,050 | ,713 | 57 | -,028 | ,839 | 57 |
| Channel_12 | ,002 | ,988 | 56 | ,063 | ,646 | 56 |
| Channel_13 | ,012 | ,929 | 56 | ,110 | ,420 | 56 |
| Channel_14 | -,175 | ,201 | 55 | -,068 | ,623 | 55 |
| Channel_15 | -,055 | ,684 | 57 | ,112 | ,407 | 57 |
| Channel_16 | -,060 | ,661 | 56 | -,063 | ,646 | 56 |
| Channel_17 | -,058 | ,670 | 56 | -,077 | ,573 | 56 |
| Channel_18 | ,025 | ,853 | 58 | ,163 | ,222 | 58 |
| Channel_19 | ,123 | ,365 | 56 | ,208 | ,124 | 56 |
| Channel_20 | ,113 | ,406 | 56 | ,113 | ,408 | 56 |

*. Correlation is significant at the 0.05 level (2-tailed).

**. Correlation is significant at the 0.01 level (2-tailed).
