# Supplementary material for: Mutual interaction between motor cortex activation and pain in fibromyalgia: EEG-fNIRS study
Source: PLoS One. 2020 Jan 23;15(1):e0228158. doi: 10.1371/journal.pone.0228158 (PMC6977766; doi:10.1371/journal.pone.0228158)
Supplement: S12 Table — (DOCX) [file pone.0228158.s012.docx]

**S12 Table. Correlations for FFT + LASER ON THE LEFT HAND condition.**

| **Correlations in FFT + LASER ON THE LEFT HAND** | | | | | | |
| --- | --- | --- | --- | --- | --- | --- |
|  | **ΔHbO_2_** | | | **ΔHb** | | |
|  | SPEED | | | SPEED | | |
| Channel | Pearson Correlation | Sig.  (2-tailed) | N | Pearson Correlation | Sig.  (2-tailed) | N |
| Channel_1 | ,045 | ,738 | 58 | -,045 | ,740 | 58 |
| Channel_2 | ,053 | ,691 | 58 | ,109 | ,415 | 58 |
| Channel_3 | ,105 | ,436 | 57 | ,231 | ,084 | 57 |
| Channel_4 | ,246 | ,063 | 58 | ,155 | ,245 | 58 |
| Channel_5 | ,075 | ,576 | 58 | ,189 | ,156 | 58 |
| Channel_6 | ,215 | ,106 | 58 | ,256 | ,052 | 58 |
| Channel_7 | ,109 | ,419 | 57 | ,017 | ,899 | 57 |
| Channel_8 | ,078 | ,563 | 58 | ,208 | ,117 | 58 |
| Channel_9 | -,018 | ,892 | 57 | ,304 | ,021 | 57 |
| Channel_10 | ,328 | ,014 | 56 | ,317 | ,017 | 56 |
| Channel_11 | ,050 | ,710 | 57 | ,090 | ,507 | 57 |
| Channel_12 | ,058 | ,670 | 56 | ,036 | ,793 | 56 |
| Channel_13 | ,092 | ,502 | 56 | ,199 | ,141 | 56 |
| Channel_14 | ,078 | ,572 | 55 | ,116 | ,400 | 55 |
| Channel_15 | ,147 | ,276 | 57 | ,134 | ,320 | 57 |
| Channel_16 | ,080 | ,560 | 56 | ,057 | ,676 | 56 |
| Channel_17 | ,031 | ,822 | 56 | ,015 | ,910 | 56 |
| Channel_18 | ,187 | ,161 | 58 | ,290 | ,027 | 58 |
| Channel_19 | ,299 | ,025 | 56 | ,203 | ,134 | 56 |
| Channel_20 | ,211 | ,119 | 56 | ,206 | ,128 | 56 |

*. Correlation is significant at the 0.05 level (2-tailed).

**. Correlation is significant at the 0.01 level (2-tailed).
