# Supplementary material for: Mutual interaction between motor cortex activation and pain in fibromyalgia: EEG-fNIRS study
Source: PLoS One. 2020 Jan 23;15(1):e0228158. doi: 10.1371/journal.pone.0228158 (PMC6977766; doi:10.1371/journal.pone.0228158)
Supplement: Attachment — Submitted filename: Response to Reviewers.docx [file pone.0228158.s013.docx]

Dear reviewers,

We are pleased for your comments of this article. I attach the corrections about your suggestion.

Reviewer #1

This is an interesting study exploring the relationship between motor activation and pain processing in fibromyalgia patients. This potentially represents a key point in fibromyalgia physiopathology. Methods are sound, the writing is straightforward.
I just have some suggestions:

- Some paragraphs require a style revision since the meaning is not completely clear (471-475)
- There are other typos that need to be corrected.
- The effect of motor activation on pain processing has a specific evolutionary purpose to allow fighting in spite of pain. Is it possible that finger tapping is not interpreted by the brain as a challenging task requiring pain silencing? please comment on this point.

We thank the reviewer for his useful comments. We completely agree with your suggestion and included this concept in the revised discussion. The changes were outlined in green color.

-Another point deserving attention is the temporal evolution of motor dysfunction in fibromyalgia. Since no relation between clinical scales and fNIRS or LEPs were found in these patients, it could be speculated that the reduced motor activation is not the mere effect of chronic pain but it is a constitutional tract of the disease. To confirm this hypothesis the experimental paradigm should be applied in patients successfully treated with drugs such as duloxetine.

We have not yet concluded to evaluate patients at follow up, but it will be a matter of a future study. We integrated this in the discussion.

- In other conditions experiencing motor activation dysfunctions (i.e. stroke, aging), re-adaptive phenomena include a reduction of lateralization for the activation of ipsilateral motor pathways. Is it possible to evaluate this balancing in the present population? Does the pain cortical activation follow the same reorganization?

We computed the lateralization index and we obtained a similar mechanism for the activation on the motor areas between groups. We integrated the results and comments in the paper.

Reviewer #2:

-Line 31: FNIRS was not defined before
- In all the text Fibromyalgia should be abbreviated as FM
- Line 62: the dot after study should be eliminated
- In Experimental study design there are some concept repetitions (line 112 and line 121)
- Line 407: what does FT mean?

We thank the reviewer for his valuable suggestions. The changes were outlined in yellow color.

-Materials and method – Subject: which criteria were used for FM classification?

Thank you for the consideration of our work. We added a more detailed description of ACR 2010 criteria.
